# Identification and Comparison of Constituents of Aurantii Fructus and Aurantii Fructus Immaturus by UFLC-DAD-Triple TOF-MS/MS

**DOI:** 10.3390/molecules23040803

**Published:** 2018-03-30

**Authors:** Yang Bai, Yuying Zheng, Wenjing Pang, Wei Peng, Hao Wu, Hongliang Yao, Panlin Li, Wen Deng, Jinle Cheng, Weiwei Su

**Affiliations:** 1 Guangdong Engineering & Technology Research Center for Quality and Efficacy Reevaluation of Post-Market Traditional Chinese Medicine, School of Life Sciences, Sun Yat-sen University, Guangzhou 510275, China; white1504@163.com (Y.B.); vicky_0224@126.com (Y.Z.); pangwenjing91@126.com (W.P.); pweiyu929@126.com (W.P.); wuhao_cpu@126.com (H.W.); yhlsysu@126.com (H.Y.); liplin3@mail.sysu.edu.cn (P.L.); 2 Guangdong Key Laboratory of Plant Resources, School of Life Sciences, Sun Yat-sen University, Guangzhou 510275, China; 3 Key Laboratory of Ultrafine Granular Powder of Herbal Medicine technology and Application of state Administration, Zhongshan 528437, China; 18007601982@163.com

**Keywords:** Aurantii Fructus, Aurantii Fructus Immaturus, qualitative, quantitative, UFLC-DAD-Triple TOF-MS/MS

## Abstract

Although Aurantii Fructus (AF) and Aurantii Fructus Immaturus (AFI) are both the fruits of the same rutaceae plant at different stages of growth, they exert similar yet distinct clinical effects. The chemical composition is crucial for quality control as well as therapeutic application. To address this concern, it is significant to evaluate the similarities and differences of the constituents in both AF and AFI. The extract of AF and AFI were comprehensively analyzed by ultra fast liquid chromatography-photodiode array detector-triple-time of flight-tandem mass spectrometry (UFLC-DAD-Triple TOF-MS/MS). Among the 40 compounds detected, 19 metabolites were detected in both the AF and AFI; whereas 13 compounds were only detected in AF and five constituents were exclusively detected in AFI. In particular, even in AFI, three compounds were only identified in AFI (*Citrus aurantium*’ L. and its cultivar). Among the 18 compounds confirmed by standard database, 13 compounds were reported in AF and AFI for the first time. Furthermore, the distinction was also revealed by the content of naringin, hesperidin, neohesperidin, and synephrine. The study directly contributed to the similarities and differences of AF and AFI. Herein, similarities and the differences in chemical profiles of AF and AFI could explain the current clinical applications.

## 1. Introduction 

Aurantii Fructus (AF) and Aurantii Fructus Immaturus (AFI) are commonly applied medicinal herbs in Traditional Chinese Medicine (TCM) practice for thousands of years. In fact, AF and AFI are both the fruits of the same rutaceae plant. AF, harvested in July, is the dried mature fruit of *Citrus aurantium’* L. and its cultivar, while AFI is the dried immature fruit of *Citrus aurantium*’ L. and its cultivar, or *Citrus sinensis* (L.) Osbeck collected from May to June. AF and AFI are collected at different stages of fruit growth with diverse clinical efficacy, thus are recorded in the Chinese Pharmacopoeia as two distinct medicinal materials [1]. According to TCM theory, AF and AFI each have their own unique clinical applications. Although AF and AFI have common effects of regulating visceral functions, AF is always used to alleviate chest pain and improve gastrointestinal functions such as alleviating dyspepsia in a gentle yet efficient manner. AFI, compared to AF, expresses a relatively more rapid and robust way of action and is often employed to disperse severe abdominal distention and to eliminate phlegm etc. Herein, it is of great interest to investigate the underlying compounds present in AF and AFI collected from the same plant source that exhibit diverse pharmacological and clinical effects. 

It is well-known that the efficacy of herbal medicines is significantly correlated with chemical composition. Thus, to compare the similarities and differences of AF/AFI comprehensively, it is necessary to evaluate their chemical composition in larger scale and accuracy. Some publications on the similarities and differences between AF and AFI have mainly focused on the quantification of several flavonoids or alkaloids by high-performance liquid chromatography-photodiode array detector (HPLC-DAD) or high-performance liquid chromatography-mass spectrometry (HPLC-MS), and the comparison of characteristic compounds in a discriminating model [2,3,4,5,6,7,8,9]. Even if HPLC-DAD and HPLC-MS have been utilized to quantify constituents, they can hardly be used to identify unknown compounds. Moreover, nuclear magnetic resonance (NMR) has been used to identify unknown components in AFI after separation [10,11]. However, to the best of our knowledge, NMR has not been applied to determine multiple constituents of AF and AFI simultaneously. Recently, high-resolution mass spectrometry, including ion trap mass spectrometry (ITIMS) and time of flight-mass spectrometry (TOF-MS) have been applied to comprehensively compare the chemical composition in AF or AFI [12,13,14]. Nevertheless, high-resolution mass spectrometry alone cannot determine new chemical structures and reference standards must be used to confirm constituents. 

In this study, the chemical composition of AF and AFI was systematically evaluated with reference standards and standard database by UFLC-DAD-Triple TOF-MS/MS. The chemical similarities and differences between AF, AFI (*Citrus aurantium*’ L. and its cultivar) and AFI (*Citrus sinensis* (L.) Osbeck) were summarized. Furthermore, the distinction was also revealed by hierarchical cluster analysis (HCA) based on the characteristics of the content of naringin, hesperidin, neohesperidin and synephrine. The differences in the above chemical compositions probably explain different clinical effects. 

## 2. Results and Discussion

### 2.1. Qualitative Comparison of Constituents in Aurantii Fructus (AF) and Aurantii Fructus Immaturus (AFI) 

**Figure 1:**
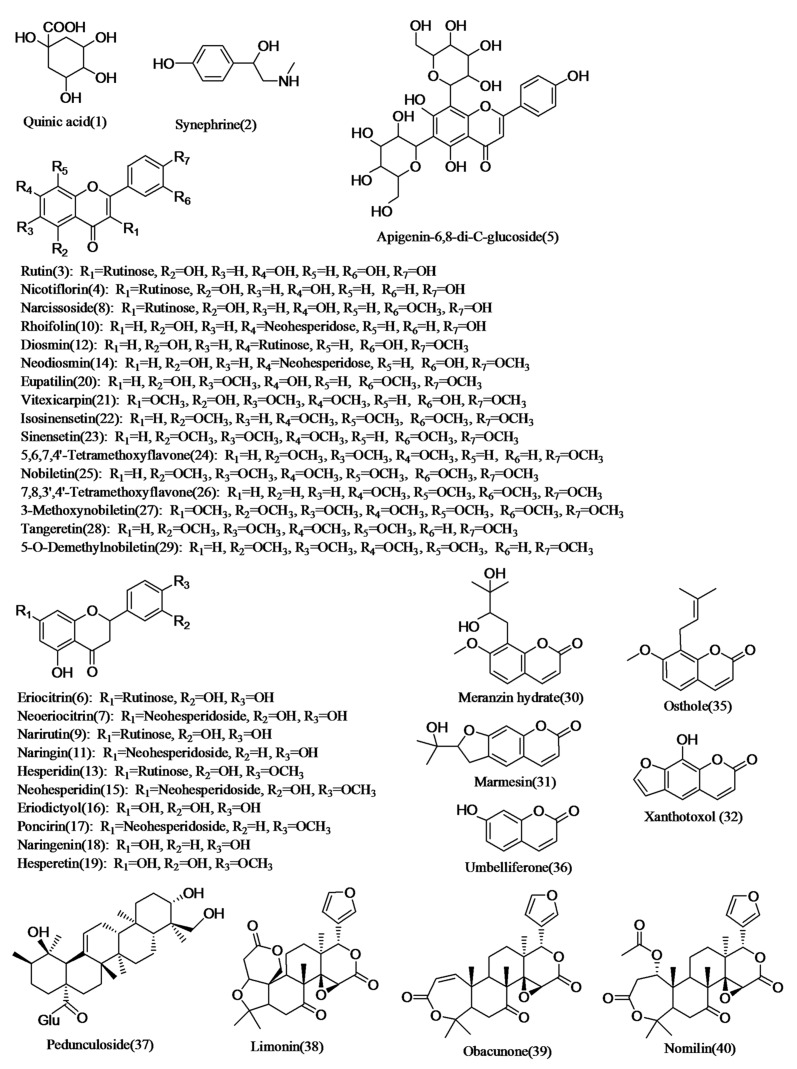
Chemical structures of compounds detected in Aurantii Fructus (AF) and Aurantii Fructus Immaturus (AFI).

**Figure 2:**
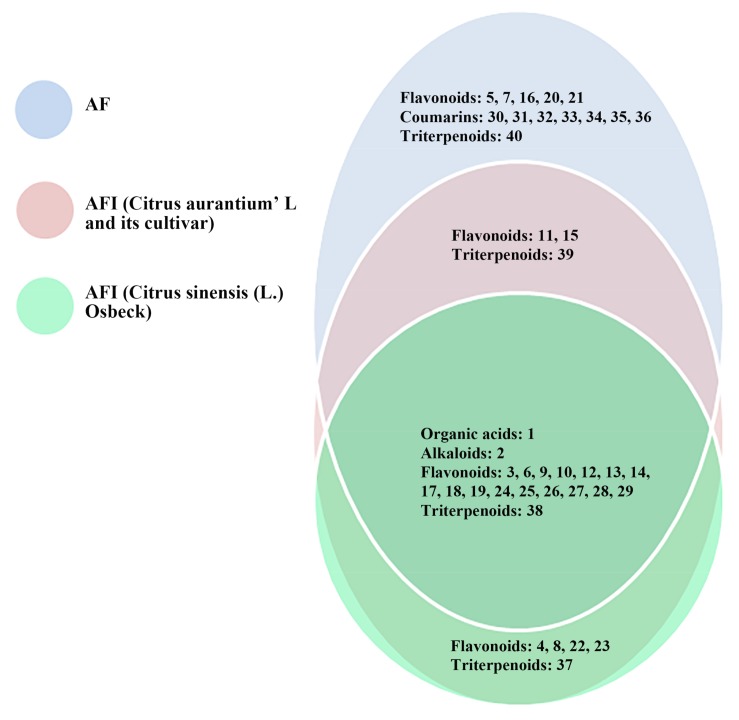
The chemical similarities and differences between AF and AFI.

In this study, a standard database (AB SCIEX LibraryView, Version 1.0) was used to identify the compounds including accurate mass-to-charge ratios, the ratio between isotopic peaks, and product ion spectra [15]. Through the database, compounds could be discriminated from their structural isomers. As summarized in Table S1, 40 compounds including 27 flavonoids, seven coumarins, four triterpenoids, an organic acid, and an alkaloid were detected (shown in Figure 1). Except for 11 standards, 18 compounds were identified in AF and AFI by standard database. Among the 18 compounds, 13 compounds were identified and reported in AF and AFI for the first time. Among the 13 compounds, limonin was detected in both AF and AFI; obacunone was detected in both AF and AFI (*Citrus aurantium*’ L. and its cultivar); nicotiflorin, narcissoside, and pedunculoside were only detected in AFI; and apigenin-6,8-di-C-glucoside (vicenin-2), eupatilin, vitexicarpin, marmesin, xanthotoxol, an isomer of xanthotoxol, osthole, and nomilin were solely detected in AF (shown in Figure 2).

### 2.2. Characterization of Organic Acids

Compound **1** (*t*_R_ = 2.54 min) generated an [M − H]^−^ ion at *m*/*z* 191, and fragment ions at *m*/*z* 127 [M − H − 18 − 18 − 28]^−^, 109 [M − H − 18 − 18 − 18 − 28]^−^, and 93 [M − H − 18 − 18 − 18 − 44]^−^ in the MS_2_ experiment, where the loss of H_2_O, carbon oxide (CO), and carbon dioxide (CO_2_) suggested the existence of a polyhydroxy compound containing carboxylic acid groups. Compound **1** was identified as quinic acid by comparing information with the standard database.

### 2.3. Characterization of Alkaloids

Compound **2** (*t*_R_ = 13.59 min) produced an [M + H]^+^ ion at *m*/*z* 168, and fragment ions at *m*/*z* 150 [M + H − 18]^+^, 135 [M + H − 18 − 15]^+^, 119 [M + H − 18 − 31]^+^, 107 [M + H − 18 − 15 − 28]^+^, and 91 [M + H − 18 − 31 − 28]^+^ where the losses of H_2_O, methyl groups (CH_3_), methylamine (CH_3_NH_2_), and carbon oxide (CO) suggested the existence of hydroxyl, phenolic hydroxyl, and methylamine groups. Fragment ions at *m*/*z* 77, 65 were the characteristic fragmentation of benzene. Based on the similarity in retention time and fragmentation patterns with reference standard, compound **2** was confirmed as synephrine.

### 2.4. Characterization of Flavonoids

**Figure 3:**
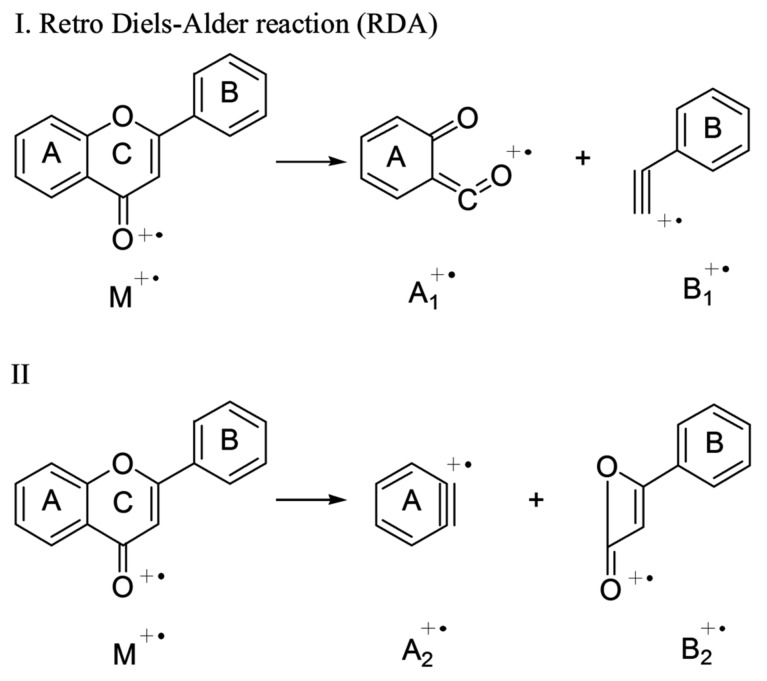
The fragmentation pathway of flavonoids.

The flavonoids in AF and AFI included flavones, flavonols, dihydroflavone, and their corresponding glycosides. The flavonoid glycosides tended to generate [M − H]^−^ ions more than [M + H]^+^ ions. After losing the glycosyl moiety, their major characteristic fragment ions could be observed as A_1_, B_1_, A_2_, and B_2_ as shown in Figure 3. In addition, the intensity of A_1_, B_1_, A_2_, and B_2_ was always higher than the others. Relative fragmentation pathways of flavonoids are presented in Figure 3. Furthermore, flavonoids tended to lose 28 Da (CO), 18 Da (H_2_O) and 15 Da (CH_3_), suggesting the existence of phenolic hydroxyl and methyl groups.

Compound **3** (*t*_R_ = 7.49 min), 4 (*t*_R_ = 7.73 min), 10 (*t*_R_ = 8.29 min) all produced [M + H]^+^ ions and [M − H]^−^ ions, respectively. Compounds **3**, **4**, and **10** similarly generated fragment ions at *m*/*z* [M + H − 308]^+^ in ESI (+) and *m*/*z* [M − H − 308]^−^ in ESI (−) by losses of rutinose. Compounds **3**, and **10** were identified as rutin and rhoifolin, respectively, with similarities in retention time and fragmentation patterns of reference standards. Compound **4** was confirmed as nicotiflorin in accordance with the standard database. Nicotiflorin has been found in AFI for the first time. 

Compound **5** (*t*_R_ = 7.50 min) produced the [M + H]^+^ ion at *m*/*z* 595 and [M − H]^−^ ion at *m*/*z* 593. We obtained fragment ions at *m*/*z* 577 [M + H − 18]^+^, 457 [M + H − 18 − 120]^+^, 295 [M + H − 18 − 120 − 162]^+^ in ESI(+), and 473 [M − H − 120]^−^, 383 [M − H − 120 − 90]^−^, 353 [M − H − 120 − 120]^−^ in ESI(−), indicating the fragmentation of glucose. Compound **5** was tentatively identified as apigenin-6,8-di-C-glucoside (vicenin-2) through a comparison with the data of Yang et al. [16]. Although Yang et al. found it in ponkan peel (harvested in November), we first detected it in AF.

Compound **6** (*t*_R_ = 7.04 min) and Compound **7** (*t*_R_ = 7.58 min) both displayed [M − H]^−^ ions at *m*/*z* 595. After the release of rutinoses (308 Da) or neohesperidoses (308 Da), their fragment ions were all observed at *m*/*z* 287. Then, their major fragment ions were all obtained at *m*/*z* 151 and 135 as A_1_ and B_1_ as shown in Figure 3. In addition, Compound **7** produced *m*/*z* 459 due to the loss of 136 Da (C_8_H_8_O_2_, B_1_ in Figure 3). Compound **6** was tentatively identified as eriocitrin by comparing the information from previous references [3,5,12], and Compound **7** was confirmed as neoeriocitrin by comparing information in the standard database. 

Although Compounds **8** (*t*_R_ = 8.18 min), **12** (*t*_R_ = 8.44 min), **13** (*t*_R_ = 8.50 min), **14** (*t*_R_ = 8.58 min) and **15** (*t*_R_ = 8.72 min) produced different [M − H]^−^ ions, their fragment ions were all displayed at *m*/*z* [M − H − 308]^−^ and [M − H − 308 − 15]^−^, suggesting the existence of rutinoses and methyl groups (CH_3_). Moreover, Compounds **12** and **14** produced [M + H]^+^ ions at *m*/*z* 609 and fragment ions at *m*/*z* 301 [M + H − 308]^+^, 286 [M + H − 308 − 15]^+^; Compounds **13** and **15** exhibited similar [M + H]^+^ ions at *m*/*z* 611 and fragment ions at *m*/*z* 303 [M + H − 308]^+^ with different *t*_R_. Compound **8** was identified as a narcissoside when compared with the standard database. Compounds **12** and **14** were tentatively identified as diosmin and neodiosmin by a comparison to the data of Zeng et al. and Shi et al. [5,12]. Compounds **13** and **15** were confirmed as hesperidin and neohesperidin through comparison with the similarity in retention time and fragmentation patterns with the reference standard. For the first time, we reported narcissoside in AFI.

Compounds **9** (*t*_R_ = 8.08 min), and **11** (*t*_R_ = 8.30 min) generated similar [M + H]^+^ ions at *m*/*z* 581 and [M − H]^−^ ions at *m*/*z* 579. We observed different percent intensity of product ions in the spectrum, indicating different chemical structures though they all produced fragment ions at 273 [M + H − 308]^+^, 271 [M − H − 308]^−^,153 [M + H − 308 − 120]^+^, 151 [M − H − 308 − 120]^−^ that attributed to losses of B_1_ (in Figure 3) and glycosyls (rutinoses for **9** or neohesperidoses for **11**). Compound **9** was identified as narirutin through a comparison with the standard database. Compound **11** was confirmed as naringin by the similarity in retention time and fragmentation patterns with the reference standard. 

Compound **16** (*t*_R_ = 11.08 min) provided [M − H]^−^ ion at *m*/*z* 287, fragment ions at *m*/*z* 151 and 135. The fragment ions were obtained as 151 Da (A_1_) and 135 Da (B_1_) shown in Figure 3. Compound **16** was identified as eriodictyol by comparing the information with the standard database.

Compound **17** (*t*_R_ = 10.72 min) showed [M + H]^+^ ion at *m*/*z* 595 and [M − H]^−^ ion at *m*/*z* 593. The fragment ions were at *m*/*z* 287 [M + H − 308]^+^ in ESI (+) and 285 [M – H − 308]^−^, 270 [M − H − 308 − 15]^−^ in ESI (−), corresponding to the losses of the neohesperidose and methyl groups. The fragment ions were at *m*/*z* 473 [M − H − 120]^−^, 387 [M − H − 216]^−^, and 327 [M − H − 266]^−^, suggesting that neohesperidose fragmented. Compound **17** was tentatively identified as poncirin by comparing it to the data of Li et al. and Shi et al. [3,12]. 

Compound **18** (*t*_R_ = 12.90 min) displayed the [M + H]^+^ ion at *m*/*z* 273 and [M − H]^−^ ion at *m*/*z* 271. They produced characteristic ions at *m*/*z* 153 [M + H − 120]^+^ in ESI (+) and 151 [M − H − 120]^−^, 119 [M − H − 152]^−^ in ESI (−), corresponding to the loss of 120 Da (B_1_) and 152 Da (A_1_) as shown in Figure 3. Furthermore, fragment ions at *m*/*z* 147 [M + H − 108 − 18]^+^, 119 [M + H − 108 − 18 − 28]^+^, and 91 [M + H − 164 − 18]^+^ were also observed, suggesting the loss of 164 Da (B_2_), 108 Da (A_2_), 28 Da (CO), and 18 Da (H_2_O), which is displayed in Figure 3. Compound **18** was confirmed as naringenin by comparing information with the standard database.

Compound **19** (*t*_R_ = 13.56 min) produced [M + H]^+^ ions at *m*/*z* 303 and [M − H]^−^ ions at *m*/*z* 301. The fragment ions were at *m*/*z* 177 [M − H − 108 − 18]^+^, 153 [M + H − 150]^+^ in ESI (+) and 286 [M − H − 15]^−^, 151 [M − H − 150]^−^ in ESI (−) by the fragmentation of the flavonoid C ring and losses of the methyl groups, A_2_ (108 Da) and B_1_ (150 Da) as shown in Figure 3. Compound **19** was confirmed as hesperetin by comparing information in the standard database.

Compound **20** (*t*_R_ = 10.54 min) produced [M − H]^‒^ ion at *m*/*z* 343 and fragment ions at *m*/*z* 328 [M − H − 15]^−^, 313 [M − H − 15 − 15]^−^, 298 [M − H − 15 − 15 − 15]^−^, and 270 [M − H − 15 − 15 − 15 − 28]^−^ by the losses of the methyl (CH_3_) and carbon oxide (CO) groups suggested the existence of phenolic hydroxyl and three methyl groups. Compound **20** was identified as eupatilin by comparing the information with the standard database. Eupatilin was first found in AF.

Compound **21** (*t*_R_ = 10.70 min) showed [M + H]^+^ ions at *m*/*z* 375 and [M − H]^−^ ions at *m*/*z* 373. There were abundant fragment ions at *m*/*z* 345 [M + H − 15 − 15]^+^, 317 [M + H − 15 − 15 − 28]^+^, 299 [M + H − 15 − 15 − 28 − 18]^+^ in ESI(+) and 358 [M − H − 15]^−^, 343 [M − H − 15 − 15]^−^, 328 [M − H − 15 − 15 − 15]^−^, 313 [M − H − 15 − 15 − 15 − 15]^−^, 257 [M − H − 15 − 15 − 15 − 15 − 28 − 28]^−^ in ESI(−) by the losses of H_2_O, methyl (CH_3_) and carbon oxide (CO) groups, suggesting the existence of phenolic hydroxyl and four methyl groups. Compound **21** was confirmed as vitexicarpin by comparing the information with the standard database. We first identified vitexicarpin in AF.

Compounds **22** (*t*_R_ = 14.71 min) and **23** (*t*_R_ = 16.10 min) both produced [M + H]^+^ ions at *m*/*z* 373 and fragment ions at *m*/*z* 358 [M + H − 15]^+^, 343 [M + H − 15 − 15]^+^, and 153 [M + H − 15 − 15 − 28 − 162]^+^. In comparison to the fragment ions of Compound **22** at *m*/*z* 315 [M + H − 15 − 15 − 28]^+^, 181 [M + H − 15 − 15 − 162]^+^, those of Compound **23** were at *m*/*z* 329 [M − 15 − 28]^+^ and 312 [M + H − 15 − 15 − 31]^+^. Some fragment ions indicated the loss of 162 Da (B_1_) as presented in Figure 3. Compounds **22** and **23** were tentatively identified as isosinensetin and sinensetin through comparison with the data of Chuang et al. and Shi et al. [6,17], respectively.

Compound **24** (*t*_R_ = 16.14 min) and **26** (*t*_R_ = 17.97 min) both generated [M + H]^+^ ions at *m*/*z* 343 and fragment ions at *m*/*z* 313 [M + H − 15 − 15]^+^, 181 [M + H − 162]^+^, 153 [M + H − 162 − 28]^+^. In contrast to the fragment ions of Compound **24** at *m*/*z* 328 [M + H − 15]^+^, 285 [M + H − 15 − 15 − 28]^+^, those of Compound **26** were at *m*/*z* 327 [M − 15]^+^, 299 [M − 15 − 28]^+^, and 282 [M + H − 15 − 15 − 31]^+^, indicating the distinction of chemical structures. Combined with the previous report [18], Compounds **24** and **26** were tentatively identified as 5,6,7,4′-tetramethoxyflavone and 7,8,3′,4′-tetramethoxy flavone, separately.

Although Compounds **25** (*t*_R_ = 14.69 min), **27** (*t*_R_ = 18.70 min), **28** (*t*_R_ = 19.48 min) exhibited different [M + H]^+^ ions at *m*/*z* 403, *m*/*z* 433 and *m*/*z* 373, respectively, they all produced [M + H − 15]^+^, [M + H − 15 − 15]^+^, and [M + H − 15 − 15 − 18]^+^, suggesting the existence of hydroxyl and methyl groups. In contrast to Compound **27**, Compounds **25** and **28** both generated fragment ions at [M + H − 15 − 15 − 15]^+^, [M + H − 15 − 15 − 15 − 28]^+^, [M + H − 15 − 15 − 18 − 28]^+^, and [M − 15 − 15 − 15 − 28 − 28]^+^. Furthermore, compound **28** presented fragment ions at [M + H − 15 − 15 − 132]^+^, and 183 [M + H − 15 − 15 − 28 − 132]^+^, indicating the loss of 132 Da (B_1_) as shown in Figure 3. Compounds **25** and **28** were confirmed as nobiletin and tangeretin, respectively, with the standard database. Based on fragment ions similarities in the previous study [18], Compound **27** was tentatively identified as 3-Methoxynobiletin.

Compound **29** (*t*_R_ = 14.93 min) produced the [M + H]^+^ ion at *m*/*z* 389 and fragment ions at *m*/*z* 374 [M + H − 15]^+^, 359 [M + H − 15 − 15]^+^, and 341 [M + H − 15 − 15 − 15]^+^ by the successive losses of methyl groups. Compared with the information in earlier studies [6,17], Compound **29** was tentatively identified as 5-*O*-Demethylnobiletin.

### 2.5. Characterization of Coumarins

Compounds **30** (*t*_R_ = 10.08 min) and **34** (*t*_R_ = 13.19 min) both generated abundant fragment ions at *m*/*z* 261 [M + H − 18]^+^, indicating dehydration. Others fragment ions of Compounds **30** and **34** were all at *m*/*z* 243 [M + H − 18 − 18]^+^, 189 [M + H − 18 − 72]^+^, 159 [M + H − 18 − 72 − 31]^+^, 131 [M + H − 18 − 72 − 31 − 28]^+^, and 103 [M + H − 18 − 72 − 31 − 28 − 28]^+^, suggesting the existence of hydroxyl, carbonyl, and methoxyl groups. The loss of 72 Da (C_4_H_8_O) was attributed to the fragmentation of the branched chain on the benzene ring after dehydration. Compound **30** was identified as meranzin hydrate with similarities in retention time and fragmentation patterns with the reference standard. Additionally, Compound **34** was identified as an isomer of meranzin hydrate through a comparison with Compound **30**.

Compound **31** (*t*_R_ = 11.13 min) provided [M + H]^+^ ion at *m*/*z* 247 and fragment ions at *m*/*z* 229 [M + H − 18]^+^, 175 [M + H − 72]^+^, and 147 [M + H − 72 − 28]^+^, demonstrating the losses of H_2_O and carbon oxide (CO) groups. The loss of 72 Da (C_4_H_8_O) was caused by the fragmentation of the branched chain on the dihydrofurane ring. For the first time, Compound **31** was confirmed as marmesin in AF, when compared to the information in the standard database.

Compounds **32** (*t*_R_ = 11.26 min) and **33** (*t*_R_ = 13.07 min) both produced [M + H]^+^ ions at *m*/*z* 203 and [M − H]^−^ ions at *m*/*z* 201. There were fragment ions at *m*/*z* 147 [M + H–28–28]^+^, 91 [M + H − 28 − 28 − 28 − 28]^+^ in ESI (+) and 173 [M − H − 28]^−^, 145 [M − H − 28 − 28]^−^, 117 [M − H − 28 − 28 − 28]^−^ in ESI (−), presenting the successive losses of carbon oxide (CO) groups. Compounds **32** and **33** were first identified as xanthotoxol and its isomer in AF when compared to the information in the standard database. 

Compound **35** (*t*_R_ = 15.75 min) generated the [M + H]^+^ ion at *m*/*z* 245 and fragment ions at 189 [M + H − 56]^+^, 131 [M + H − 56 − 31 − 28]^+^, 103 [M + H − 56 − 31 − 28 − 28]^+^, and 77 [M + H − 56 − 31 − 28 − 28 − 26]^+^, responsible the losses of carbon oxide (CO) and methoxyl (CH_3_O) groups. Furthermore, the loss of 56 Da (C_4_H_8_) arose from the fragmentation of the branched chain on the benzene ring. Comparing the information in the standard database, compound **35** was identified as osthole for the first time in AF.

Compound **36** (*t*_R_ = 28.84 min) produced the [M + H]^+^ ion at *m*/*z* 163 and fragment ions at 119 [M + H − 44]^+^, 107 [M + H − 28 − 28]^+^, 91 [M + H − 44 − 28]^+^, and 77 [M + H − 44 − 42]^+^ by the losses of carbon oxide (CO) and carbon dioxide (CO_2_) groups. Compound **36** was confirmed as umbelliferone by comparing the information in the standard database.

### 2.6. Characterization of Triterpenoids

Compound **37** (*t*_R_ = 14.20 min) presented the [M − H]^−^ ion at *m*/*z* 649 and fragment ion at 487 [M − H − 162]^−^, attributed to the loss of glucose (Glu). Compound **37** was identified as pedunculoside when compared to the information in the standard database. For the first time, pedunculoside was identified in AFI.

**Figure 4:**
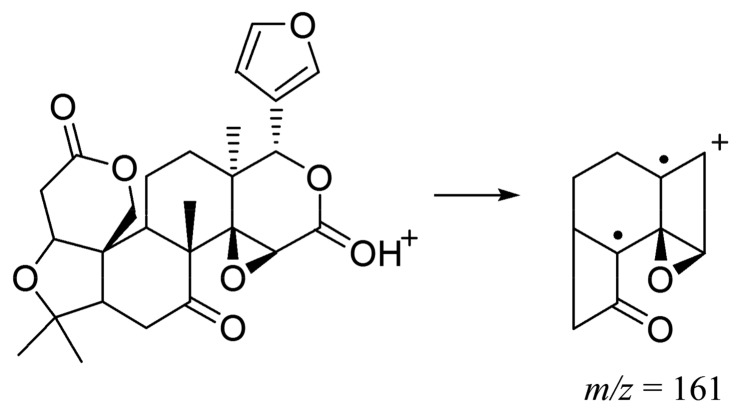
The fragmentation of limonoids.

Although Compounds **38** (*t*_R_ = 16.37 min), **39** (*t*_R_ = 16.20 min), and **40** (*t*_R_ = 17.78 min) exhibited different [M + H]^+^ ions and [M − H]^−^ ions, they all generated fragment ions at [M + H − 46]^+^ and *m*/*z* 161, suggesting the existence of a lactone group and six-membered rings of limonoids in Figure 4. Due to the distinction of chemical structures, Compound **38** showed fragment ions at *m*/*z* 367 [M + H − 46 − 15 − 15 − 28]^+^ and 339 [M + H − 46 − 15 − 15 − 28 − 28]^+^; however, Compound **39** showed fragment ions at *m*/*z* 435 [M − H − 18]^−^ and 391 [M − H − 18 − 44]^−^. In addition, the fragment ion at *m*/*z* 411 [M + H − 44 − 59]^+^ of Compound **40** was attributed to the loss of the acetoxyl group on the seven-membered ring after decarboxylation. Compounds **38**, **39**, and **40** were confirmed as limonin, obacunone, and nomilin through a comparison to the information in the standard database. Although Yang et al. found limonin and nomilin in ponkan peel (harvested in November) [16], we first identified limonin in AF and AFI, obacunone in AF and AFI (*Citrus aurantium*’ L. and its cultivar), and nomilin in AF.

### 2.7. Data Analysis 

**Figure 5:**
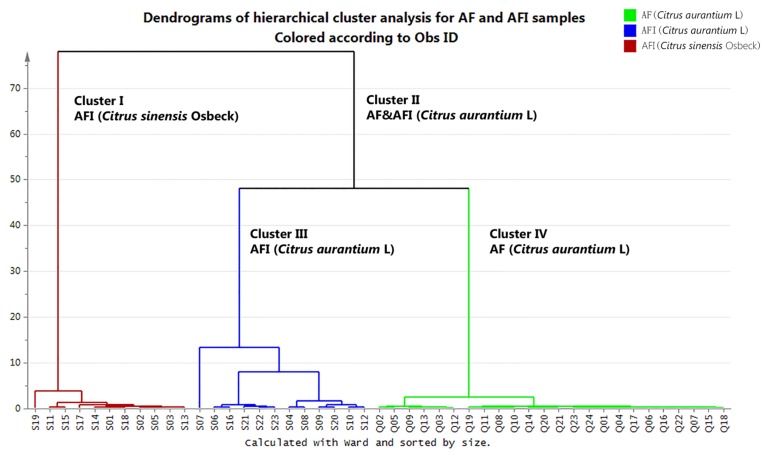
Evaluation of the differences between AF and AFI with hierarchical cluster analysis (HCA).

To evaluate the differences between AF and AFI, herarchical cluster analysis (HCA) was performed based on the characteristics of the contents of naringin, hesperidin, neohesperidin, and synephrine. The HCA results demonstrated significant differences in the form of a dendrogram calculated based on Ward’s minimum variance method (shown in Figure 5). All samples could be clearly divided into two clusters: 11 batches of AFI derived from *Citrus sinensis* Osbeck were classified into Cluster I (left); and AFI and AF derived from *Citrus aurantium* L. and its cultivar were classified into Cluster II (right). Moreover, Cluster II could be divided into two further clusters according the harvest time, while all AFI were included in Cluster III and all AF were included in Cluster IV, which was consistent with the classification in the Chinese Pharmacopoeia.

### 2.8. Methodology Validation

- Specificity: The integration peak in the chromatogram of the sample solution corresponded in time to the peak in the chromatogram of the standard solution. No such peak of that retention time appeared in the chromatogram of the solvent.
- Linearity and range: The regression analysis was performed with a peak area integral value of Y, and reference substance injection volume X. The regression equations and linearity ranges were as follows: naringin: Y = 29.655X + 0.0322, R^2^ = 1.0000, 0.0789~2.3661 μg; hesperidin: Y = 35.29X + 0.0086, R^2^ = 1.0000, 0.0077~0.2321 μg; neohesperidin: Y = 31.137X + 0.0157, R^2^ = 1.0000, 0.0826~2.4765 μg; synephrine: Y = 54.454X − 0.081, R^2^ = 1.0000, 0.0156~0.623 μg. All results above showed good linear relationship.
- Precision: The repeatability (all RSD < 2%) and the intermediate precision of contents (all RAD < 2%) indicated high precision.
- Accuracy: All mean recoveries were in the range of 95‒105% (naringin, 98.85%; hesperidin, 99.61%; neohesperidin, 99.58%; synephrine, 99.66%, all RSD < 4%), showing high accuracy.
- Stability: The analytes were found to be stable for 48 h for both samples and standards (all RSD < 2%).
- Ruggedness: The method showed good ruggedness on different columns (all RSD < 2%).

In this study, the chemical compositions were compared between AF and AFI. The 40 compounds in AF and AFI were detected and identified comprehensively by UFLC-DAD-Triple TOF-MS/MS, including 27 flavonoids, seven coumarins, four triterpenoids, an organic acid and an alkaloid. Based on 40 compounds, the 19 compounds of both AF and AFI probably related to the common effect of regulating visceral function. Moreover, chemical differences between AFI and AF were found. The five compounds found solely in AFI mostly belonged to flavonols of flavonoids, that have been shown by other studies to show anti-angiogenesis, anti-inflammation, neuroprotection, and hepatoprotective effects [19,20,21,22,23,24,25,26,27]. The 13 compounds detected only in AF included five flavonoids, seven coumarins, and a triterpenoid. With the exception of the pharmacological and clinical effects of the five compounds in AFI listed above [28,29,30,31,32,33,34,35,36,37,38,39,40,41], the 13 compounds also exerted other pharmacological activities and clinical effects. Some of them can promote osteogenesis and protect cartilage [42,43,44,45,46,47]; eupatilin and osthole can provide significant cardioprotective effects [48,49]; meranzin hydrate can promote intestinal transit and gastric emptying [50]; conversely, eupatilin can decrease human lower gastrointestinal motility [51]; eriodictyol can attenuate acute lung injury and kidney injury through its antioxidative and anti-inflammatory activity [52,53]. Hence the 13 compounds may have comprehensively exerted clinical effect of alleviating chest pain and improving gastrointestinal functions. Furthermore, AF’s moderate effects might be due to a combination of multiple constituents. Interestingly, AFI (*Citrus sinensis* (L.) Osbeck) tastes sweeter than AF and AFI (*Citrus aurantium*’ L. and its cultivar); and bitter compounds (naringin, neohesperidin and obacunone) were not detected in AFI (*Citrus sinensis* (L.) Osbeck), but only identified in AF and AFI (*Citrus aurantium*’ L. and its cultivar), indicating the difference in AFI plant species [54,55,56]. 

Moreover, the distinction between them was also revealed by the content of naringin, neohesperidin, hesperidin, and synephrine. The HCA results clearly showed differences in chemical profiles of different plant species as well as differences in harvesting times of the same species.

The differences in chemical profiles of AF and AFI could explain the current clinical applications. Herein our study shed light on the potential pharmacological effects of these identified compounds of AF and AFI that deserves further investigation in the future. 

## 3. Materials and Methods

### 3.1. Reagents and Materials

Twelve batches of AFI (*Citrus aurantium*’ L. and its cultivar) were collected from four different districts in China (six batches from Jiangxi province; one batch from Hunan province; two batches from Sichuan province; three batches from Zhejiang province) in 2016. Eleven batches of AFI (*Citrus sinensis* (L.) Osbeck) were harvested from three different districts in China (four batches from Jiangxi province; four batches from Hunan province; three batches from Sichuan province) in 2016. Twenty-four batches of AF (*Citrus aurantium*’ L. and its cultivar) were collected from four different districts in China (ten batches from Jiangxi province; four batches from Hunan province; five batches from Sichuan province; five batches from Zhejiang province) in 2016. All samples were deposited in the Guangdong Key Laboratory of Plant Resources and identified by chief pharmacist Liwei Yang (Guangdong Institute for Food and Drug Control, Guangzhou, China). 

Naringin (purity: 94.7%), neohesperidin (purity: 99.6%), hesperidin (purity: 94.7%), synephrine (purity: 99.4%), rutin (purity: 91.9%), rhoifolin (purity: 92.3%) were purchased from the National Institute for Control of Biological and Pharmaceutical Products of China. Naringenin (purity: 95%) was purchased from Sigma-Aldrich (St. Louis, MO, USA). Hesperetin and eriodictyol were purchased from Sinova lab with purity >95% determined by high performance liquid chromatography (HPLC). Umbelliferone and meranzin hydrate were isolated in our lab, and their structures were elucidated by NMR with a purity >98% determined by HPLC.

Methanol of HPLC grade (Honeywell, Morris Plains, NJ, USA) and formic acid of HPLC grade (Sigma-Aldrich) were used. All water used was distilled and further purified by a Milli-Q system (Millipore, Milford, MA, USA). Other reagents used in the experiment were analytical grade.

### 3.2. Standard Solutions and Sample Preparation 

The mixed solution of 11 standards for identification was prepared in 50% methanol at the concentration of 10 μg /mL for each compound. After 0.2 g of powdered sample was accurately weighed to a 100 mL glass-stoppered conical flask, 50 mL of 50% methanol (methanol:water = 1:1, *v*:*v*) was added. The filled flask was weighed with a precision of ±0.01 g, sonicated for 30 min (250 W, 40 kHz), allowed to cool, and adjusted to the initial weight by 50% methanol as needed. After taking the filtrate 10 mL precisely to 25 mL volumetric flask, 50% methanol was add to the mark. Then, the solution was filtered with a membrane filter (0.45 μm) to collect the successive filtrate. The filtrate was used as the sample solution.

### 3.3. Analysis by UFLC-DAD-Triple TOF-MS/MS

A sample solution was prepared using four methods including sonication in methanol (0.5 h), reflux by methanol (1.5 h), sonication in 50% methanol (methanol:water = 1:1, *v*:*v*) (0.5 h) and reflux by 50% methanol (1.5 h). Separately the sample solution was injected to determine the amount of naringin (%), hesperidin (%), and neohesperidin (%). The method of sonication in 50% methanol for 30 min was chosen in the experiment since its efficiency was the greatest. After the optimization of LC and MS conditions, a simple UFLC-DAD-Triple TOF-MS/MS method was developed.

Analysis was performed with a Shimadzu UFLC XR instrument (Shimadzu Corp., Kyoto, Japan) equipped with an in-line degasser, a binary pump, an autosampler, a column oven, and a photodiode array detector (DAD). Separation was carried out on a Phenomenex C_18_ column (150 mm × 3.0 mm, 3 μm) at 40 °C. The mobile phase consisted of acetonitrile (A) and 0.1% formic acid (*v*/*v*) in water (B) using a linear gradient 30% A (0–5 min), 30 to 80% A (5–27 min), 80% to 100%A (27–28 min), 100%A (28–33 min), 100% to 10%A (33–34 min), and 10%A (34–40 min). The injected volume was 5 μL with the flow rate kept at 0.3 mL/min. The DAD detector scanned from 190 to 400 nm. 

Detections were performed by a hybrid triple quadrupole time-of-flight mass spectrometer (AB SCIEX Triple TOF™ 5600 plus, AB Sciex, Foster City, CA, USA) equipped with electrospray ionization (ESI) source, analyst software (PeakView, Version 2.1, AB Sciex, Shanghai, China) and a standard database (LibraryView, Version 1.0, AB Sciex). The standard database included information on 1213 compounds (molecular formula, name, accurate mass-to-charge ratios, the ratio between isotopic peaks and product ion spectrum). The standards in the standard database were purchased from the National Institute for Control of Biological and Pharmaceutical Products of China. After opening the data files in the software, all the compound information could be imported from the standard database. Then, the software automatically calculated similarity score. With an error less than 5 ppm and a score greater than 50% as screening criterion, 40 compounds were chosen to analyze.

The TOF-MS worked in a full scan mode and mass range was set at *m*/*z* 50–1200 in both positive and negative ion modes. The acquisition of MS/MS data was accomplished by the IDA (information-dependent acquisition) mode. The conditions of the mass spectrometer were as follows: ion source gas1 55 psi; ion source gas2 55 psi; curtain gas 35 psi; temperature 550 °C; ion spray voltage floating 5500 V (positive) or −4500 V (negative); collision energy 40 V and declustering potential 60 V. Nitrogen was used as the nebulizer and auxiliary gas. 

The contents of naringin, hesperidin, neohesperidin and synephrine were defined as four characteristics in the analysis to differentiate and classify the AF and AFI samples. Hierarchical cluster analysis (HCA) of samples was performed by SIMCA-P software (version 13.0, Umetrics, Malmö, Sweden) to evaluate the differences between AF and AFI. After the contents of the four compounds in 24 batches of AF and 23 batches of AFI formed a dataset (shown in Table S3), the dataset was imported to the software. Then, the distances between 47 samples were calculated based on Ward’s minimum variance method, and the results was presented as a dendrogram.

### 3.4. Methodology Validation

Methodology validation was established according to the Guidelines for the investigation and formulation of Chinese material medica monographs in Chinese Pharmacopeia 2015.

Specificity: Separately, an equal volume of the blank solvent, standard solution (naringin, neohesperidin, hesperidin, and synephrine) and sample solution (AF and AFI) were injected into the chromatograph, and the chromatograms were recorded. 

Linearity and range: The standard solution (naringin: 78.87 μg/mL, hesperidin: 7.738 μg/mL, neohesperidin: 82.55 μg/mL) was injected with 1 μL, 2 μL, 5 μL, 10 μL, 15 μL, 20 μL, 25 μL, and 30 μL. The standard solution of synephrine (30.15 μg/mL) was injected with 0.5 μL, 1 μL, 2 μL, 5 μL, 10 μL, 15 μL, and 20 μL. 

Precision: Repeatability was assessed by injecting six sample solutions of the same batch into the instrument to calculate their contents. Intermediate precision was evaluated by preparing sample solutions independently in duplicate with the same sample by two operators, on two separate days, and injected into different instruments to calculate their contents, separately. 

Accuracy: Sample solutions were prepared in three different amount levels (low, medium and high) for triplicate experiments at each level. The differences of the found amount and original amount divided by the spiked amount of naringin, hesperidin, neohesperidin, and synephrine were used to calculate the recovery. 

Stability: The stability of the sample solution and standard solution was investigated. It was carried out by comparing the peak areas of naringin, hesperidin, neohesperidin, and synephrine in the chromatograph of the same sample solution and standard solution, after being stored at room temperature for different times (0, 2, 4, 6, 8, 12, 24, 48 h). Stability was evaluated by calculating the relative standard deviation (RSD) of area obtained. 

Ruggedness: The ruggedness of the established method was evaluated by examining its stability with small variations of procedural parameters; the effects of different columns (Welch Ultimate XB-C_18_, 4.6 mm × 25 cm, 5 μm; Agilent Zorbax Eclipse Plus C_18_, 4.6 mm × 25 cm, 5 μm; Elite Hypersil ODS_2_, 4.6 mm × 25 cm, 5 μm) on the relative retention time of each characteristic peak and their content were investigated. 

## Supplementary Materials

The following are available online. Table S1: Qualitative comparison of constituents in AF and AFI; Table S2: Qualitative information of constituents in AF and AFI; Table S3: Content of naringin, hesperidin, neohesperidin and synephrine in AF and AFI on the dried basis (*n* = 2, RSD < 2%).
