# Supplementary material for: Identification and Comparison of Constituents of Aurantii Fructus and Aurantii Fructus Immaturus by UFLC-DAD-Triple TOF-MS/MS
Source: Molecules. 2018 Mar 30;23(4):803. doi: 10.3390/molecules23040803 (PMC6017871; doi:10.3390/molecules23040803)
Supplement: Supplementary file 1 [file molecules-23-00803-s001.pdf]

Table S1. Qualitative comparison of constituents in AF and AFI.

		No.	t_R/ min	Molecular formula	Identification
Compounds in AF and AFI	Organic acids	1	2.54	$C_7H_{12}O_6$	Quinic acid
	Alkaloids	2	13.59	$C_9H_{13}NO_2$	Synephrine
	Flavonoids	3	7.49	$C_{27}H_{30}O_{16}$	Rutin
		6	7.04	$C_{27}H_{32}O_{15}$	Eriocitrin
		9	8.08	$C_{27}H_{32}O_{14}$	Narirutin
		10	8.29	$C_{27}H_{30}O_{14}$	Rhoifolin
		12	8.44	$C_{28}H_{32}O_{15}$	Diosmin
		13	8.50	$C_{28}H_{34}O_{15}$	Hesperidin
		14	8.58	$C_{28}H_{32}O_{15}$	Neodiosmin
		17	10.72	$C_{28}H_{34}O_{14}$	Poncirin
		18	12.90	$C_{15}H_{12}O_5$	Naringenin
		19	13.56	$C_{16}H_{14}O_6$	Hesperetin
		24	16.14	$C_{19}H_{18}O_6$	5,6,7,4'-Tetra methoxyflavone
		25	14.69	$C_{21}H_{22}O_8$	Nobiletin
		26	17.97	$C_{19}H_{18}O_6$	7,8,3',4'-Tetra methoxyflavone
		27	18.70	$C_{22}H_{24}O_9$	3-Methoxynobiletin
		28	19.48	$C_{20}H_{20}O_7$	Tangeretin
		29	14.93	$C_{20}H_{20}O_8$	5-O-Demethyl nobiletin
	Triterpenoids	38	16.37	$C_{26}H_{30}O_8$	Limonin
Compounds present in AF exclusively	Flavonoids	5	7.50	$C_{27}H_{30}O_{15}$	Vicenin-2
		7	7.58	$C_{27}H_{32}O_{15}$	Neoeriocitrin
		16	11.08	$C_{15}H_{12}O_6$	Eriodictyol
		20	10.54	$C_{18}H_{16}O_7$	Eupatilin
		21	10.70	$C_{19}H_{18}O_8$	Vitexicarpin
	Coumarins	30	10.08	$C_{15}H_{16}O_4$	Meranzin hydrate
		31	11.13	$C_{14}H_{14}O_4$	Marmesin
		32	11.26	$C_{11}H_6O_4$	Xanthotoxol
		33	13.07	$C_{11}H_6O_4$	Isomer of xanthotoxol
		34	13.19	$C_{15}H_{16}O_4$	Isomer of meranzin hydrate
		35	15.75	$C_{15}H_{16}O_3$	Osthole
		36	28.84	$C_9H_6O_3$	Umbelliferone
	Triterpenoids	40	17.78	$C_{28}H_{34}O_9$	Nomilin
Compounds present in AFI exclusively	Flavonoids	4	7.73	$C_{27}H_{30}O_{15}$	Nicotiflorin
		8	8.18	$C_{28}H_{32}O_{16}$	Narcissoside
		22	14.71	$C_{20}H_{20}O_7$	Isosinensetin
		23	16.10	$C_{20}H_{20}O_7$	Sinensetin
	Triterpenoids	37	14.20	$C_{36}H_{58}O_{10}$.	Pedunculoside
Compounds in AF and AFI (*Citrus aurantium'* L and its cultivar)	Flavonoids	11	8.30	$C_{27}H_{32}O_{14}$	Naringin
		15	8.72	$C_{28}H_{34}O_{15}$	Neohesperidin
	Triterpenoids	39	16.20	$C_{26}H_{30}O_7$	Obacunone

Table S2. Qualitative information of constituents in AF and AFI.

No.	[M+H]+/ppm (Error)	[M-H]−/ppm (Error)	Main fragment ions (*m/z*) Positive ion mode	Negative ion mode	Identification
1		191.0563 (–0.8)		127.0403, 109.0298, 93.0359	Quinic acid
2	168.1020 (–1.9)		150.0915, 135.0682, 119.0498, 107.0509, 91.0556, 77.0407, 65.0420		Synephrine
3	611.1581 (6.5)	609.1891 (0.9)	303.0471	301.0742	Rutin
4	595.1637 (–0.8)	593.1517 (0.8)	287.0527	285.0412	Nicotiflorin
5	595.1658 (0.1)	593.1517 (0.8)	577.1536, 457.1071, 295.0563	473.1096, 383.0778, 353.0679	Vicenin-2
6		595.1666 (–0.4)		287.0555, 151.0045, 135.0446	Eriocitrin
7		595.1663 (–0.9)		459.1155, 287.0570, 151.0041,135	Neoeriocitrin
8		623.1619 (0.2)		315.0514, 300.0270	Narcissoside
9	581.1863 (–1.0)	579.1716 (–0.7)	273.0771, 153.0155	459.1139, 271.0603, 151.0037	Narirutin
10	579.1688 (–1.2)	577.1592 (0.5)	271.0583	269.0457	Rhoifolin
11	581.1845 (–1.0)	579.1747 (0.4)	273.0710, 153.0127	459.1180, 271.0622, 151.0041	Naringin
12	609.1790 (–3.9)	607.1671 (0.4)	301.0686, 286.0460	299.0573, 284.0349	Diosmin
13	611.1946 (–0.8)	609.1834 (0.8)	303.0864	301.0723, 286.0476	Hesperidin
14	609.1792 (–3.6)	607.1671 (0.4)	301.0686, 286.0452	299.0560, 284.0326	Neodiosmin
15	611.1952 (–0.8)	609.1874 (0.8)	303.0867	301.0727, 286.0495	Neohesperidin
16		287.0563 (0.7)		151.0034, 135.0442	Eriodictyol
17	595.2002 (–0.6)	593.1922 (–0.7)	287.0899	473.1457, 387.1073, 327.0864,285.0768, 270.0532	Poncirin
18	273.0758 (0.1)	271.0630 (1.0)	153.0191, 147.0463, 119.0513, 91.0551	151.0051, 119.0498	Naringenin
19	303.0865 (0.5)	301.0723 (1.8)	177.0514, 153.0172	286.0442, 164.0109, 151.0035,136.0155	Hesperetin
20		343.0827 (1)		328.0572, 313.0346, 298.0118, 270.0152	Eupatilin
21	375.1073 (–0.4)	373.0932 (0.8)	345.0559, 317.0626, 299.0523	358.0693, 343.0453, 328.0264, 313.0009, 257.0137	Vitexicarpin
22	373.1285 (0.8)		358.1067, 343.0833, 315.0873, 181.0173,153.0174		Isosinensetin
23	373.1282 (0)		358.1045, 343.0823, 329.1067, 312.1003, 153.0198		Sinensetin
24	343.1174 (–0.7)		328.0948, 313.0695, 285.0711, 181.0135,153.0188		5,6,7,4'-Tetramethoxyflavone
25	403.1388 (0.1)		388.1132, 373.0872, 358.0646, 355.0780, 330.0712, 327.0837,301.0678		Nobiletin
26	343.1174 (–0.8)		327.0826, 313.0689, 299.0908, 282.0860, 181.0110, 153.0185		7,8,3',4'-Tetramethoxyflavone
27	433.1496 (0.7)		418.1235, 403.0985, 385.0897		3-Methoxynobiletin
28	373.1284 (–0.7)		358.1065, 343.0827, 328.0560, 325.0731, 300.0646,		Tangeretin

			297.0788, 271.0647, 211.0260, 183.0299		
29	389.1232 (—0.2)		374.1003, 359.0738, 341.0637		5-O-Demethyl nobiletin
30	261.1121 (—0.1)		243.1004, 189.0543, 159.0453, 131.0499, 103.0547		Meranzin hydrate
31	247.0964 (—0.5)		229.0831, 175.0378, 147.0448		Marmesin
32	203.0334 (–2.6)	201.0193 (5.4)	147.0432, 91.0555	173.0237, 145.0292, 117.0351	Xanthotoxol
33	203.0318 (-2.6)	201.0201 (4.0)	147.0435, 91.0553	173.0231, 145.0289, 117.0333	Isomer of Xanthotoxol
34	261.1121 (–0.3)		243.1029, 189.0551, 159.0447, 131.0520, 103.0587		Isomer of meranzin hydrate
35	245.1174 (0.6)		189.0538, 131.0503, 103.0573, 77.0457		Osthole
36	163.0391 (0.8)		119.0541,107.0536, 91.0607, 77.0451		Umbelliferone
37	649.4019 (0.9)			695.4019, 487.3434	Pedunculoside
38	471.2011 (-0.5)	469.1863 (–1.0)	425.1964, 367.1906, 339.1957,161.0600	229. 1241	Limonin
39	455.2068 (0.8)	453.1918 (-0.3)	409.2301, 161.0599	435.1800, 391.1908	Obacunone
40	515.2273 (–0.4)		469.2255, 411.2170, 161.0599		Nomilin

Table S3. Contents of naringin, hesperidin, neohesperidin, and synephrine in AF and AFI on the dried basis (n = 2, RSD < 2%).

	No.	Province	Naringin (%)	Hesperidin (%)	Neohesperidin (%)	Synephrine (%)		No.	Province	Naringin (%)	Hesperidin (%)	Neohesperidin (%)	Synephrine (%)
AF	Q01	Jiangxi	4.27	0.28	3.39	0.008	AFI (*Citrus aurantium'* L and its cultivar)	S04	Jiangxi	9.59	0.73	15.48	0.49
	Q02	Jiangxi	5.90	0.68	4.97	0.039		S06	Jiangxi	9.52	0.36	7.81	—
	Q03	Jiangxi	5.99	0.55	5.20	0.019		S07	Jiangxi	5.46	0.88	22.98	1.18
	Q04	Jiangxi	4.46	0.29	3.34	0.020		S08	Jiangxi	7.14	0.58	15.76	0.63
	Q05	Jiangxi	4.90	0.62	3.74	0.030		S09	Jiangxi	11.41	0.54	11.52	0.44
	Q06	Jiangxi	4.93	0.31	3.83	0.007		S10	Jiangxi	11.81	0.42	13.98	0.55
	Q07	Jiangxi	4.77	0.34	3.90	0.012		S12	Hunan	13.52	0.51	13.44	0.56
	Q08	Jiangxi	5.25	0.38	4.17	0.024		S16	Sichuan	8.60	0.39	10.52	—
	Q09	Jiangxi	6.54	0.46	5.30	0.057		S20	Sichuan	10.52	0.22	10.30	0.60
	Q10	Jiangxi	5.55	0.39	4.49	0.046		S21	Zhejiang	10.46	0.19	12.53	—
	Q11	Hunan	5.36	0.29	2.97	0.224		S22	Zhejiang	10.86	0.44	12.96	—
	Q12	Hunan	6.21	0.58	5.03	0.018		S23	Zhejiang	10.44	0.44	11.70	—
	Q13	Hunan	5.15	0.31	2.76	0.335	AFI (*Citrus sinensis* (L.) Osbeck)	S01	Jiangxi	—	0.94	—	0.32
	Q14	Hunan	5.82	0.43	0.21	0.031		S02	Jiangxi	—	0.81	—	0.29
	Q15	Sichuan	5.06	0.33	3.61	0.013		S03	Jiangxi	—	0.46	—	0.86
	Q16	Sichuan	5.06	0.31	3.77	0.015		S05	Jiangxi	—	0.96	—	0.22
	Q17	Sichuan	4.06	0.21	3.40	0.099		S11	Hunan	—	0.97	—	0.52
	Q18	Sichuan	5.10	0.33	3.63	0.012		S13	Hunan	—	0.62	—	0.60
	Q19	Sichuan	5.36	0.18	4.78	0.169		S14	Hunan	—	0.92	—	0.46
	Q20	Zhejiang	4.18	0.35	3.35	0.012		S15	Hunan	—	0.73	—	0.91
	Q21	Zhejiang	4.52	0.37	3.27	0.012		S17	Sichuan	—	0.70	—	0.21
	Q22	Zhejiang	4.73	0.34	3.44	0.013		S18	Sichuan	—	0.85	—	0.47
	Q23	Zhejiang	4.99	0.14	2.91	0.139		S19	Sichuan	—	0.84	—	1.33
	Q24	Zhejiang	4.24	0.14	2.56	0.154							

Note: "—": The constituent was not detected.
